# Evolution of Xenotransplantation as an Alternative to Shortage of Donors in Heart Transplantation

**DOI:** 10.7759/cureus.26284

**Published:** 2022-06-24

**Authors:** Ishaq J Wadiwala, Pankaj Garg, John H Yazji, Emad Alamouti-fard, Mohammad Alomari, Md Walid Akram Hussain, Mohamed S Elawady, Samuel Jacob

**Affiliations:** 1 Cardiothoracic Surgery, Mayo Clinic, Jacksonville, USA; 2 Colorectal Surgery, Mayo Clinic, Jacksonville, USA

**Keywords:** religious perspective, porcine endogenous retroviruses (perv), genetic engineering, immune rejection, potential animals, xenotransplantation

## Abstract

This review aims to show and illustrate the history, current, ethical considerations, and limitations concerning xenotransplantation. Due to the current shortage of available donor organs for transplantation, many alternative sources are being examined to solve the donor shortage. One of them is xenotransplantation which refers to the transplantation of organs from one species to another. Compared to other nonhuman primates (NHP), pigs are ideal species for organ harvesting as they rapidly grow to human size in a handful of months. There is much advancement in the genetic engineering of pigs, which have hearts structurally and functionally similar to the human heart. The role of genetic engineering is to overcome the immune barriers in xenotransplantation and can be used in hyperacute rejection and T cell-mediated rejection. It is technically difficult to use large animal models for orthotopic, life-sustaining heart transplantation. Despite the fact that some religious traditions, such as Jewish and Muslim, prohibit the ingestion of pork products, few religious leaders consider that donating porcine organs is ethical because it saves human life. Although recent technologies have lowered the risk of a xenograft producing a novel virus that causes an epidemic, the risk still exists. It has major implications for the informed consent procedure connected with clinical research on heart xenotransplantation.

## Introduction and background

The scenario with heart transplantation exemplifies the current paucity of donor organs for transplantation in patients with terminal organ failure. The need for a human heart has surpassed the number of hearts available. The transplant community has responded with a full-court push of strategies to address the supply-demand imbalance. For patients with heart failure, the best treatment is a heart transplant from a deceased or living donor. Many alternative sources are being looked at as possible solutions to fix the lack of human-donated organs, especially in heart transplantation. In order to expand the availability of hearts from deceased donors, most Western countries have established major organ recovery programs, some voluntary and others required, with complex allocation algorithms to ensure both equity and optimal results in recipients [1]. Organs are provided to patients who are predicted to live the longest and for whom tissue matching is challenging. Donated hearts are also dispersed according to organ quality, meaning that supply will likely grow from non-traditional sources such as cardiac death donors or older donors with more comorbidity.

One possible way to close the gap between the supply and demand for a donor's heart is the use of xenotransplantation. The term xenotransplantation refers to the transplantation of organs from one species to another. Many legends exist in folklore in which one body part is replaced with that of an animal. For example, Anubis had a jackal's head, while Hindu deity Ganesh's human head was replaced with that of an elephant. Greek mythology had multiple chimeric figures, including a minotaur born with a bull's head, centaurs with human torsos and heads but the lower body resembled that of a horse, and the sphinx has the head of a woman, the haunches of a lion, and the wings of a bird [2].

**Figure 1:**
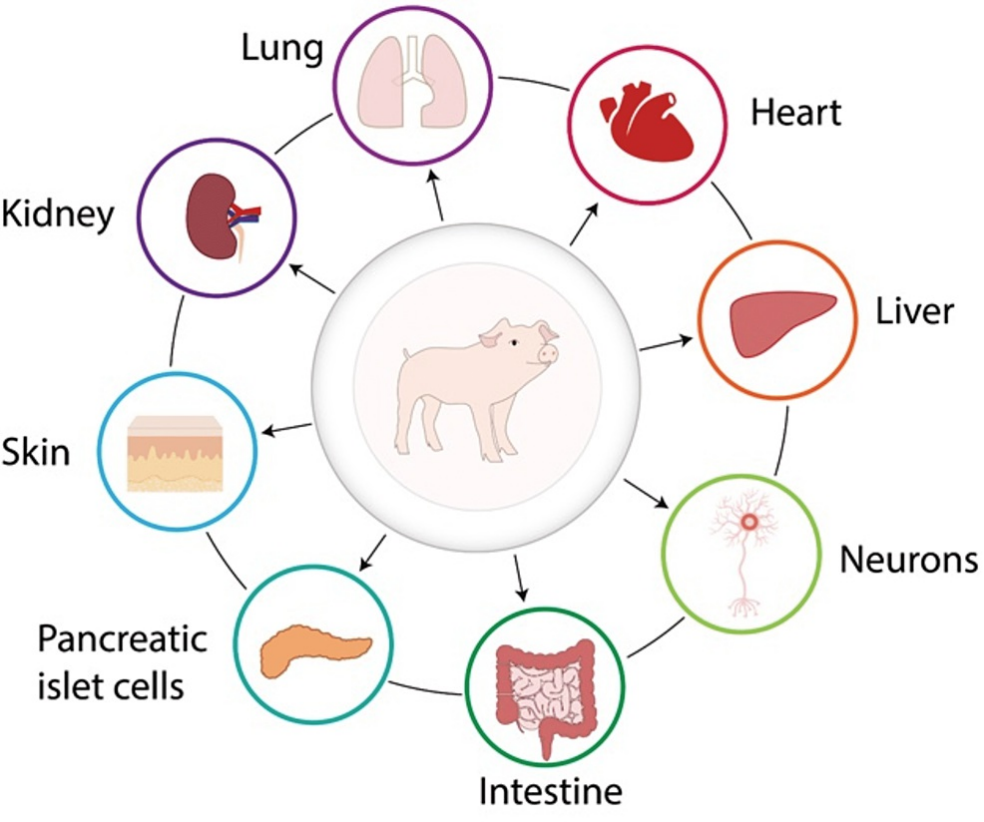
Different organs used in transplant These are the different organs that can be used in xenotransplant. Pigs' organs are suitable for xenotransplant, especially the heart. Image credit: The authors of the current study.

Because of their size and physiological similarities to humans, the low risk of xenozoonosis, and the simplicity with which genetic engineering techniques may be applied to generate rejection-resistant pig organs, pigs are regarded to be the most suited animal for xenotransplantation (Figure 1). The genetic difference between pigs and humans, on the other hand, has created challenges to xenotransplantation, such as immunological rejection and the possibility of xenozoonotic [3].

## Review

History of xenotransplantation

The history of cross-species transplant dates to the seventeenth century, with blood being transfused for various ailments, and teeth and skin grafts being used from animals into humans [4-6]. Alexis Carrel opened the door for organ transplantation by pioneering the triangulation technique of vascular anastomosis [7]. In the twentieth century, numerous attempts were conducted worldwide using nonhuman primate organs and mammals such as rabbits, sheep, and pigs. In 1906, Mathieu Jaboulay was credited with the first attempt to transplant pig and goat kidneys into patients with chronic kidney disease; both surgeries failed due to thrombosis of the grafts [8]. Ernst Unger tried in 1909 using a macaque's kidney, which ended up in a similar fate to Jaboulay’s operations [9]. The early attempts were marred by disappointing outcomes mainly due to lack of immunosuppression and were not pursued until the 1960s [3]. Keitlh Reemtsha from Tulane University used various immunosuppressive medicines and full-body radiation to graft a monkey kidney into a person, and the graft was not rejected. After two months, however, the patient died of pneumonia. Following that, Reemtsha et al. worked on several patients, the longest of whom lived for nine months [10,11].

James Hardy, a cardiac surgeon in Jackson, Mississippi, conducted the first chimp heart transplant on a 68-year-old male patient suffering from heart failure, shock, and gangrene in his left leg. He was supposed to get an orthotopic heart transplant. However, during this era, the recipient and donor had to "die" simultaneously, which did not occur, and xenotransplantation was performed. Unfortunately, the chimp's heart was not sufficient in size and eventually failed to satisfy the human body's demands, pumping for only an hour, and the patient lost consciousness. An autopsy of the chimp's heart revealed that it had refused to be transplanted [12].

In 1967, Christian Barnard transplanted the first human heart into Mr. Louis Washansky, who survived for 18 days. The second patient lasted 19 months, and the fifth and sixth recipients lasted 13 years and 24 years, respectively [13,14]. After Barnard's first heart transplant, there was a scramble to get the heart transplants done in different centers and countries, totaling over 100 heart transplants in 24 countries, putting xenotransplantation on the back burner [15]. After Bernard’s heart transplant in 1967, many mammalian hearts were used for transplant, except "Baby Fae," who suffered from hypoplastic left heart syndrome and received a baboon heart by Leonard Lee Bailey for 20 days. "Baby Fae" was the first recorded pediatric heart transplant in history (Table 1).

**Table 1:** History of xenotransplanted hearts All xenotransplanted hearts that are done in the past are listed here. This table reflects the time, surgeon, location, and survival of the hearts transplanted [16-23].

Year	Surgeon (Location)	Donor Organ and Source	Survival	References
1964	James Hardy (USA)	Chimpanzee heart	90 minutes	[12]
1968	Donald Ross (UK)	Pig heart	4 minutes	[16]
1968	Denton Cooley (USA)	Sheep heart	10 minutes	[17]
1969	Pierre Marion (France)	Chimpanzee heart	“quickly”	[18]
1977	Christian Barnard (South Africa)	Chimpanzee heart	4 days	[19]
1984	Leonard Bailey (USA)	Baboon heart	20 days	[20]
1992	Zbigniew Religa (Poland)	Pig heart	23 hours	[21]
1996	Dhaniram Baruah (India)	Pig heart	7 days	[22]
2022	Bartley Griffith (USA)	Pig heart	60 days	[23,24]

Current role of xenotransplantation

In January 2022, a genetically modified pig heart transplant was done on David Bennet Sr, who was suffering from terminal heart failure, by Dr. Barley P. Griffith, Dr. Muhammad M. Mohiuddin, and the University of Maryland Medical Center team. The patient's condition had worsened to the point where he needed extracorporeal membrane oxygenation (ECMO). Furthermore, due to disobedience to medication and doctor's recommendations, he was not a candidate for a human heart transplant or left ventricular assist device (LVAD) therapy due to an erratic cardiac rhythm. This patient was not part of a randomized clinical trial; thus, the medical team needed the Food and Drug Administration (FDA) approval to allow a one-time exception for "compassionate use." The patient progressed gradually; he could watch TV, communicate with family, and work with a physical therapist, but his condition deteriorated, and he died exactly two months after receiving his transplant [24].

After a while, Dr. Griffith and his team were able to make a breakthrough by executing the first pig heart transplant that lasted two months, eight times longer than the previous attempt. The pig’s heart used was heavily genetically modified; altogether, 10 genes were altered [25]. First, the galactose α-l,3-galactose (Gal) was knocked out, creating an engineered α1,3-galactosyltransferase gene-knockout (GTKO) pigs. Then, human complement regulatory proteins (CRPs), to prevent the body's complement from attacking the pig tissue, three clusters of differentiation (CD), 46 membrane cofactor proteins, CD55 (decay-accelerating factor), and CD59 (membrane attack complex inhibitor protein) were added to Gal [26,27]. The addition of human antithrombotic genes to the GTKO/CRP pigs prevented thrombotic microangiopathy. Finally, various immunotherapeutic medications were administered to reduce T cells and B cells and inhibit cardiac muscle hypertrophy [28,29]. The experimental drug KPL-404 inhibits cross-communication between B cells and T cells by attaching to CD40, resulting in decreased antipig antibodies [25]. A perioperative cardiac xenograft dysfunction (PCXD) is a deterrent to a successful transplant. Usually, graft failure is seen within the two days after the transplant, and better preservation solutions and techniques, namely the ex vivo device heart storage, have led to greater survival and decreased PCXD incidence [30].

**Figure 2:**
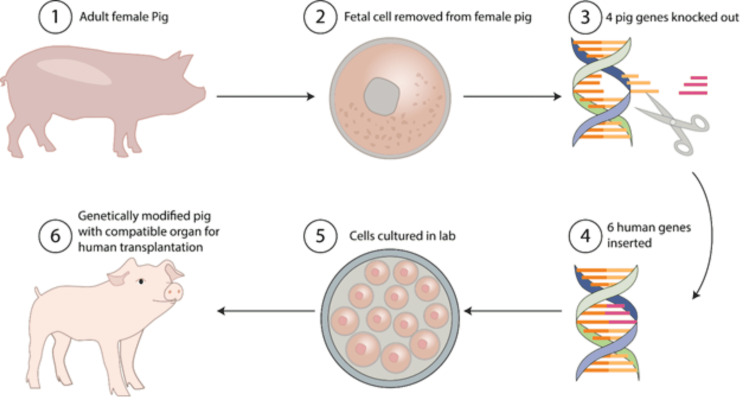
Genetically modified pigs The process of cloning an adult female pig by culturing and then modifying a few genes (knock-in and knock-out). The final cells are cultivated to form a mature pig whose organs are ready for transplantation. Image credit: The authors of the current study.

The role of genetic engineering is to overcome the immune barriers to use xenotransplantation in hyperacute rejection and T cell-mediated rejection. It is technically difficult to use large animal models for orthotopic, life-sustaining heart transplantation. Most cardiac xenografts are heterotopic, which means the graft is attached to the recipient's large blood vessels in the abdominal cavity and serves as an appendage. These cardiac xenografts are not functional hearts. With long-term immunosuppression, heart grafts having heterotopic porcine GalT KO, human CRP, and thrombomodulin transgenic genes have survived in baboons for several years [28]. More recently, orthotopic heart xenotransplantation has advanced with life-sustaining graft survival rates exceeding six months with GalT KO, human CD46, and human thrombomodulin pig hearts transplanted into baboons treated with rituximab, antithymocyte globulin (ATG), anti-CD40/CD40L, mycophenolate mofetil, and steroids (Figure 2) [29].

Transgenic production of hA20, a zinc finger protein enzyme triggered by tumor necrosis factor (TNF), inhibits nuclear factor B activation and TNF-mediated apoptosis. Although there was no discernible increase in organ survival, expression of this transgene in pigs was confined to the heart, where it appeared to somewhat reduce ischemia-perfusion damage [31]. Human thrombomodulin (CD141), an anticoagulant protein expressed on the surface of endothelial cells (ECs), was transgenically produced. It was one of the pig heart transgenic changes thought to be important in facilitating the long-term survival of heterotopic heart xenografts in baboons [32].

According to Mohiuddin et al., GTKO.hCD46.hTBM pig heart xenografts heterotopically transplanted into a baboon survived for more than a year. The immunomodulatory approach used anti-CD40 antibody, which resulted in decreased antipig antibody production, thrombotic microangiopathy in the graft, and incidence of systemic consumptive coagulopathy [33].

Another study by Mohiuddin et al. tested an iterative modification of a previous immunomodulation method, one that predominantly targets the anti-CD154-CD40 costimulation pathway, in baboon recipients of GTKO.hCD46.hTBM porcine hearts. It shows that continuing anti-CD40 (2C10R4) antibody treatment prevents xenograft rejection, resulting in graft survival of more than two years [34]. Something similar may be explored in pig-to-human xenotransplantation.

Pigs are regarded to be the best species for donating organs for human heart transplantation because they are easy to raise, mature quickly, reach an adult human size in a matter of months, and have cardiac anatomy that is similar to that of humans in size and function (Table 2) [35].

**Table 2:** Comparison between pig and baboon as donors The advantages and disadvantages of the pig as a potential source of organs and cells for humans, in contrast with those of the baboon in this role, are shown in this table.

	Pig	Baboon
Availability	Unlimited	Limited
Breeding potential	Good	Poor
Period to reproductive maturity	4–8 months	3–5 years
Length of pregnancy	114 ± 2 days	173–193 days
Number of offspring	5–12	1–2
Growth	Rapid (adult human size within 6 months)	Slow (9 years to reach maximum size)
Size of adult organs	Adequate	Inadequate
Cost of maintenance	Significantly lower	High
Anatomical similarity to humans	Moderately close	Close
Physiological similarity to humans	Moderately close	Close
Relationship of the immune system to humans	Distant	Close
Knowledge of tissue typing	Considerable (in selected herds)	Limited
Necessity for blood type compatibility with humans	Probably unimportant	Important
Experience with genetic engineering	Considerable	None
Risk of transfer of infection (xenozoonosis)	Low	High
Availability of specific pathogen-free animals	Yes	Yes
Public opinion	More in favor	Mixed

Religious and community perspectives

Compared to primates, pigs are ideal species for organ harvesting as they rapidly grow to human size in a handful of months. There is much advancement in the genetic engineering of pigs, which have hearts structurally and functionally similar to the human heart [36]. Multiple zoonotic, ethical, moral, and even religious issues surrounding xenotransplants are present [37]. Social acceptance of xenotransplants is not always clear as demonstrated by the example of Dhaniram Baruah, who was sentenced to 40 days in prison. An outraged crowd wrecked his lab and farm, and the town mocked and shunned him [38]. Religious concerns arose in the Jewish and Islamic faiths, which historically forbid the use of pigs in any form. However, there is some reconciliation when the procedure is used to save a life or avoid worsening a disease.

Despite the fact that some religious traditions, such as Jewish and Muslim, prohibit the use of pork products, other religious leaders consider that donating porcine organs is acceptable because it saves human life [38]. Dietary restrictions imposed on Jews and Muslims do not affect tissue implantation. Vegetarianism is practiced by about half of Seventh-day Adventists and more than a third of Buddhists, which may translate into a refusal to use xenogeneic tissue. Many Hindus outright forbid using human tissue and animal products, while others accept donations of human organs and tissue. Vegans may prefer allogeneic tissue to xenogeneic tissue. Scientologists, Baptists, Lutherans, Evangelicals, and Catholics accept allogeneic and xenogeneic acellular grafts. Methodists, Jehovah's Witnesses, and The Church of Jesus Christ of Latter-Day Saints allow individuals to make their own decisions [39].

One possible answer to these problems is clarifying that just a fraction of all transplant patients might benefit from xenotransplantation. On the other hand, others may benefit from therapy methods that do not include the use of animals. As a result, the justification for using animals in this manner would be that just a small number of animals are required as organ donors because they are the only viable alternative for a subset of patients in need of organs [40].

Animal rights groups have resisted using animals as a source of organs because of the animal intelligence, sentience, and propensity for suffering. The issue of donor animals having rights is controversial. Animal rights activists consider pigs to be "complex, intellectual beings," who believe pigs should not be slain for human organs although they have been utilized as a source of meat for generations [41]. Like other nonhuman primates, baboons exhibit sophisticated social behaviors, and their usage raises several ethical considerations. Pigs, on the other hand, are significantly less contentious. People for the Ethical Treatment of Animals (PETA) stated that they were "opposed to the use of animals and animal tissues for experimentation, medical training, and clinical treatments including the use of biological meshes" in response to the use of animal-derived xenogeneic biologic meshes for soft tissue repairs [42].

Transmission of infection

The spread of swine infections to human recipients is a major concern in the field of xenotransplantation. HIV/AIDS, Ebola, bird flu (influenza A/H5N1), and swine flu (influenza A/H1N1) are examples of disease transfer from animal to human. Many viral pathogens, such as hepatitis E virus, cytomegaly virus, and swine lymphotropic herpesviruses, can be found in pigs. The majority of problems can be avoided by breeding without pathogens [31]. However, because porcine endogenous retroviruses (PERV) are present in the genomes of all pigs, they cannot be eradicated through pathogen-free breeding. It is a major concern that swine endogenous retroviruses could infect human cells, mutate, cause cancer, or mix with other viruses to create new infectious diseases. Recent advances, such as the gene-editing deletion of all proviruses in the pig genome, have made these possibilities less likely but not impossible [41]. Also, many retroviruses are endogenous to pigs, which have the possibility of spreading to human cells and causing diseases; this is partially tackled using the gene-knockout technique, which considerably reduces the risk [42].

PERVs are classified as PERV-A, PERV-B, and PERV-C [43]. All pig breeds carry PERV-A and PERV-B, but PERV-C is only found in a few [44]. PERV-A/C, a recombinant virus, had higher infectivity in human cells [45]. As a result, the International Xenotransplantation Association recommends that the donor pig for xenotransplantation be free of PERV-C [46]. PERV transmission from pig to human and human to human cells was discovered in several in vitro studies [47]. However, only certain cell types were infected as PERVs are unable to infect some cell types due to the lack of a functioning receptor on the majority of cell surfaces [48]. Niu et al. exploited somatic cell nuclear transfer to inactivate all PERVs in a swine primary cell line and raise healthy PERV-deficient pigs in 2017 [49]. These pigs offer a novel approach to xenotransplantation that reduces the risk of PERVs. The susceptibility of these pigs to PERV reinfection, however, is unknown. Even though the CRISPR/Cas9 PERV-inactivated cell line PK15 produced defective virus particles, they were no longer infectious. The mutant PK15 cells are protected from reinfection by the PERV [50].

Although recent technologies have reduced the risk of a xenograft producing a novel virus that causes an epidemic, the risk still exists, and it has significant implications for the informed consent procedure associated with heart xenotransplantation clinical research. Transplant recipients who receive a swine heart endanger themselves, their families, and the general public's health. As a result, as required by US Public Health Service rules for xenotransplantation, the informed consent process must thoroughly communicate those hazards in detail. As many zoonoses (e.g., HIV and Creutzfeldt-Jakob disease) have year-long latency periods before being discovered, the patient must be aware of the need for lifelong clinical and laboratory monitoring for xenogeneic diseases as well as certain invasions of privacy, such as mandatory sexual contact reporting. The need for various forms of necessary follow-up, including the requirement for an autopsy at the time of death, should be adequately communicated to family and other close contacts [51].

David Bennett

Doctors discovered symptoms of a virus known as "porcine cytomegalovirus" in David Bennett, a 57-year-old man with heart failure, who survived for two months after receiving the first-ever pig heart transplant in January 2022. He appeared to be in good health for a few weeks, but after around 40 days, his condition deteriorated, and he died on March 8 [52]. According to Roni Caryn Rabin of the New York Times, the donor pig was grown by the biotech company Revivicor (Blacksburg, Virginia). It had been genetically modified to reduce the risk of rejection by Bennett's body and was checked for infections multiple times. Virus transmission from animals to people is a worry for such transplants. According to the Massachusetts Institute of Technology, if a virus adapts inside a patient and spreads to doctors and nurses, it might cause a new pandemic. However, experts believe the virus detected in Bennett's donor is incapable of infecting human cells. Despite Bennett's death, the transplant was deemed a success, and doctors hope to continue researching how to save lives using animal organs.

Preservation of the transplanted organ

Antegrade flushing of the coronary arteries with a cardioplegic solution followed by ischemic storage at 4°C is the gold standard for clinical heart transplantation [53]. Ischemia lasting less than four hours is associated with a higher survival probability, but ischemia lasting more than six hours is associated with a higher risk of mortality within a year. Greater organ protection is required to reduce cardiac metabolism and prevent ischemia-reperfusion harm [54].

Steen et al. reported successful orthotopic allotransplantation of pig hearts after 24 hours of preservation. They perfused donor hearts with an oxygenated 8°C cold hyperoncotic cardioplegic nutritional solution comprising hormones and erythrocytes and used a heart preservation system. Before being transplanted into a recipient pig, the grafts were kept in a cardiac preservation system and perfused with the same solution for additional 24 hours [55]. When the first pig-to-human transplantation was done earlier this year, something similar was done during organ storage and preservation. There they modified the technique developed by Lund University where they used an ex vivo device to store the heart once removed from the pig. Then the heart was immersed in a fluid media that included hormones such as adrenaline and cortisol and dissolved cocaine. This showed good preservation of the heart with good graft function after transplantation into the patient [25].

While standard ischemic cardioplegia solutions have been used with great success in human allotransplantation for many years, the current studies indicate that they are insufficient for pig hearts transplanted into baboons, according to Längin et al. Ischemic storage led to significant deterioration of heart function and decreased tissue oxygen delivery in more than half of the xenotransplantation trials, resulting in multi-organ failure. On the other hand, cold nonischemic heart preservation with continuous perfusion reliably prevented early graft failure. Long-term preclinical observations after heart xenotransplantation necessitate stable perioperative survival [56].

Future of xenotransplantation

Through a combination of many discoveries in enhanced immunosuppression and genetic engineering as well as a better understanding of cross-species incompatibilities, the field of xenotransplantation has made significant progress in the last decade. As a result, cardiac xenograft survival has been demonstrated for more than three years [57]. With the correct immunosuppressive regimen, xenograft survival can be extended. Acute humoral xenograft rejection, a delayed form of antibody-mediated rejection, is a rare event, and acute cellular rejection is not the predominant cause of graft failure. A coagulation problem between the recipient and the donor appears to be the cause of graft failure. When a pig heart is transplanted, this problem presents as thrombotic microangiopathy. This results in ischemic damage to the myocardium, which can lead to consumptive coagulopathy [58]. The future of xenotransplantation is bright.

## Conclusions

Xenotransplantation is a technique that can be used in the future, but it is fraught with complications. This innovation has the potential to reduce, if not eliminate, the waiting list. Using xenotransplantation could be the solution to increasing the heart donor pool. However, numerous problems must be answered before xenotransplantation is considered a therapeutic practice rather than an experimental procedure. When asked about the future of xenotransplantation, Sir Roy Calne stated that it "is just around the corner, but it may be a very long corner." Other researchers have also helped shorten the corner and make xenotransplantation one step closer to reality.
